# Supplementary material for: Streptococcus thermophilus alters the expression of genes associated with innate and adaptive immunity in human peripheral blood mononuclear cells
Source: PLoS One. 2020 Feb 11;15(2):e0228531. doi: 10.1371/journal.pone.0228531 (PMC7012395; doi:10.1371/journal.pone.0228531)
Supplement: S1 Table — (DOCX) [file pone.0228531.s001.docx]

**Table S1**

| **Position** | **Uni**  **gene** | **Symbol** | **Description** | **GENE name** |
| --- | --- | --- | --- | --- |
| A01 | Hs.507080 | APCS | Amyloid P component, serum | HEL-S-92n/PTX2/SAP |
| A02 | Hs.529053 | C3 | Complement component 3 | AHUS5/ARMD9/ASP/C3a/C3b/CPAMD1/HEL-S-62p |
| A03 | Hs.2490 | CASP1 | Caspase 1, apoptosis-related cysteine peptidase (interleukin 1, beta, convertase) | ICE/IL1BC/P45 |
| A04 | Hs.303649 | CCL2 | Chemokine (C-C motif) ligand 2 | GDCF-2/HC11/HSMCR30/MCAF/MCP-1/MCP1/SCYA2/SMC-CF |
| A05 | Hs.514821 | CCL5 | Chemokine (C-C motif) ligand 5 | D17S136E/RANTES/SCYA5/SIS-delta/SISd/TCP228/eoCP |
| A06 | Hs.184926 | CCR4 | Chemokine (C-C motif) receptor 4 | CC-CKR-4/CD194/CKR4/CMKBR4/ChemR13/HGCN:14099/K5-5 |
| A07 | Hs.450802 | CCR5 | Chemokine (C-C motif) receptor 5 | CC-CKR-5/CCCKR5/CCR-5/CD195/CKR-5/CKR5/CMKBR5/IDDM22 |
| A08 | Hs.46468 | CCR6 | Chemokine (C-C motif) receptor 6 | BN-1/C-C CKR-6/CC-CKR-6/CCR-6/CD196/CKR-L3/CKRL3/CMKBR6/DCR2/DRY6/GPR29/GPRCY4/STRL22 |
| A09 | Hs.113222 | CCR8 | Chemokine (C-C motif) receptor 8 | CC-CKR-8/CCR-8/CDw198/CKRL1/CMKBR8/CMKBRL2/CY6/GPRCY6/TER1 |
| A10 | Hs.163867 | CD14 | CD14 molecule | - |
| A11 | Hs.631659 | CD4 | CD4 molecule | CD4mut |
| A12 | Hs.472860 | CD40 | CD40 molecule, TNF receptor superfamily member 5 | Bp50/CDW40/TNFRSF5/p50 |
| B01 | Hs.592244 | CD40LG | CD40 ligand | CD154/CD40L/HIGM1/IGM/IMD3/T-BAM/TNFSF5/TRAP/gp39/hCD40L |
| B02 | Hs.838 | CD80 | CD80 molecule | B7/B7-1/B7.1/BB1/CD28LG/CD28LG1/LAB7 |
| B03 | Hs.171182 | CD86 | CD86 molecule | B7-2/B7.2/B70/CD28LG2/LAB72 |
| B04 | Hs.85258 | CD8A | CD8a molecule | CD8/Leu2/MAL/p32 |
| B05 | Hs.709456 | CRP | C-reactive protein, pentraxin-related | PTX1 |
| B06 | Hs.1349 | CSF2 | Colony stimulating factor 2 (granulocyte-macrophage) | GMCSF |
| B07 | Hs.632586 | CXCL10 | Chemokine (C-X-C motif) ligand 10 | C7/IFI10/INP10/IP-10/SCYB10/crg-2/gIP-10/mob-1 |
| B08 | Hs.198252 | CXCR3 | Chemokine (C-X-C motif) receptor 3 | CD182/CD183/CKR-L2/CMKAR3/GPR9/IP10-R/Mig-R/MigR |
| B09 | Hs.190622 | DDX58 | DEAD (Asp-Glu-Ala-Asp) box polypeptide 58 | RIG-I/RIGI/RLR-1/SGMRT2 |
| B10 | Hs.2007 | FASLG | Fas ligand (TNF superfamily, member 6) | ALPS1B/APT1LG1/APTL/CD178/CD95-L/CD95L/FASL/TNFSF6 |
| B11 | Hs.247700 | FOXP3 | Forkhead box P3 | AIID/DIETER/IPEX/JM2/PIDX/XPID |
| B12 | Hs.524134 | GATA3 | GATA binding protein 3 | HDR/HDRS |
| C01 | Hs.181244 | HLA-A | Major histocompatibility complex, class I, A | HLAA |
| C02 | Hs.650174 | HLA-E | Major histocompatibility complex, class I, E | EA1.2/EA2.1/HLA-6.2/MHC/QA1 |
| C03 | Hs.643447 | ICAM1 | Intercellular adhesion molecule 1 | BB2/CD54/P3.58 |
| C04 | Hs.37026 | IFNA1 | Interferon, alpha 1 | IFL/IFN/IFN-ALPHA/IFN-alphaD/IFNA13/IFNA@ |
| C05 | Hs.529400 | IFNAR1 | Interferon (alpha, beta and omega) receptor 1 | AVP/IFN-alpha-REC/IFNAR/IFNBR/IFRC |
| C06 | Hs.93177 | IFNB1 | Interferon, beta 1, fibroblast | IFB/IFF/IFN-beta/IFNB |
| C07 | Hs.856 | IFNG | Interferon, gamma | IFG/IFI |
| C08 | Hs.520414 | IFNGR1 | Interferon gamma receptor 1 | CD119/IFNGR/IMD27A/IMD27B |
| C09 | Hs.193717 | IL10 | Interleukin 10 | CSIF/GVHDS/IL-10/IL10A/TGIF |
| C10 | Hs.845 | IL13 | Interleukin 13 | IL-13/P600 |
| C11 | Hs.41724 | IL17A | Interleukin 17A | CTLA-8/CTLA8/IL-17/IL-17A/IL17 |
| C12 | Hs.83077 | IL18 | Interleukin 18 (interferon-gamma-inducing factor) | IGIF/IL-18/IL-1g/IL1F4 |
| D01 | Hs.1722 | IL1A | Interleukin 1, alpha | IL-1A/IL1/IL1-ALPHA/IL1F1 |
| D02 | Hs.126256 | IL1B | Interleukin 1, beta | IL-1/IL1-BETA/IL1F2 |
| D03 | Hs.701982 | IL1R1 | Interleukin 1 receptor, type I | CD121A/D2S1473/IL-1R-alpha/IL1R/IL1RA/P80 |
| D04 | Hs.89679 | IL2 | Interleukin 2 | IL-2/TCGF/lymphokine |
| D05 | Hs.98309 | IL23A | Interleukin 23, alpha subunit p19 | IL-23/IL-23A/IL23P19/P19/SGRF |
| D06 | Hs.73917 | IL4 | Interleukin 4 | BCGF-1/BCGF1/BSF-1/BSF1/IL-4 |
| D07 | Hs.2247 | IL5 | Interleukin 5 (colony-stimulating factor, eosinophil) | EDF/IL-5/TRF |
| D08 | Hs.654458 | IL6 | Interleukin 6 (interferon, beta 2) | BSF2/HGF/HSF/IFNB2/IL-6 |
| D09 | Hs.624 | CXCL8 | Interleukin 8 | GCP-1/GCP1/IL8/LECT/LUCT/LYNAP/MDNCF/MONAP/NAF/NAP-1/NAP1 |
| D10 | Hs.522819 | IRAK1 | Interleukin-1 receptor-associated kinase 1 | IRAK/pelle |
| D11 | Hs.289052 | IRF3 | Interferon regulatory factor 3 | - |
| D12 | Hs.166120 | IRF7 | Interferon regulatory factor 7 | IMD39/IRF-7H/IRF7A/IRF7B/IRF7C/IRF7H |
| E01 | Hs.172631 | ITGAM | Integrin, alpha M (complement component 3 receptor 3 subunit) | CD11B/CR3A/MAC-1/MAC1A/MO1A/SLEB6 |
| E02 | Hs.656213 | JAK2 | Janus kinase 2 | JTK10/THCYT3 |
| E03 | Hs.726603 | LY96 | Lymphocyte antigen 96 | ESOP-1/MD-2/MD2/ly-96 |
| E04 | Hs.524579 | LYZ | Lysozyme | LZM |
| E05 | Hs.431850 | MAPK1 | Mitogen-activated protein kinase 1 | ERK/ERK-2/ERK2/ERT1/MAPK2/P42MAPK/PRKM1/PRKM2/p38/p40/p41/p41mapk/p42-MAPK |
| E06 | Hs.138211 | MAPK8 | Mitogen-activated protein kinase 8 | JNK/JNK-46/JNK1/JNK1A2/JNK21B1/2/PRKM8/SAPK1/SAPK1c |
| E07 | Hs.499674 | MBL2 | Mannose-binding lectin (protein C) 2, soluble | COLEC1/HSMBPC/MBL/MBL2D/MBP/MBP-C/MBP1/MBPD |
| E08 | Hs.458272 | MPO | Myeloperoxidase | - |
| E09 | Hs.517307 | MX1 | Myxovirus (influenza virus) resistance 1, interferon-inducible protein p78 (mouse) | IFI-78K/IFI78/MX/MxA |
| E10 | Hs.82116 | MYD88 | Myeloid differentiation primary response gene (88) | MYD88D |
| E11 | Hs.618430 | NFKB1 | Nuclear factor of kappa light polypeptide gene enhancer in B-cells 1 | EBP-1/KBF1/NF-kB1/NF-kappa-B/NF-kappaB/NFKB-p105/NFKB-p50/NFkappaB/p105/p50 |
| E12 | Hs.81328 | NFKBIA | Nuclear factor of kappa light polypeptide gene enhancer in B-cells inhibitor, alpha | IKBA/MAD-3/NFKBI |
| F01 | Hs.159483 | NLRP3 | NLR family, pyrin domain containing 3 | AGTAVPRL/AII/AVP/C1orf7/CIAS1/CLR1.1/FCAS/FCAS1/FCU/MWS/NALP3/PYPAF1 |
| F02 | Hs.738731 | NOD1 | Nucleotide-binding oligomerization domain containing 1 | CARD4/CLR7.1/NLRC1 |
| F03 | Hs.592072 | NOD2 | Nucleotide-binding oligomerization domain containing 2 | ACUG/BLAU/CARD15/CD/CLR16.3/IBD1/NLRC2/NOD2B/PSORAS1 |
| F04 | Hs.538979 | RAG1 | Recombination activating gene 1 | RAG-1/RNF74 |
| F05 | Hs.256022 | RORC | RAR-related orphan receptor C | NR1F3/RORG/RZR-GAMMA/RZRG/TOR |
| F06 | Hs.591607 | SLC11A1 | Solute carrier family 11 (proton-coupled divalent metal ion transporters), member 1 | LSH/NRAMP/NRAMP1 |
| F07 | Hs.642990 | STAT1 | Signal transducer and activator of transcription 1, 91kDa | CANDF7/IMD31A/IMD31B/IMD31C/ISGF-3/STAT91 |
| F08 | Hs.463059 | STAT3 | Signal transducer and activator of transcription 3 (acute-phase response factor) | ADMIO/APRF/HIES |
| F09 | Hs.80642 | STAT4 | Signal transducer and activator of transcription 4 | SLEB11 |
| F10 | Hs.524518 | STAT6 | Signal transducer and activator of transcription 6, interleukin-4 induced | D12S1644/IL-4-STAT/STAT6B/STAT6C |
| F11 | Hs.272409 | TBX21 | T-box 21 | T-PET/T-bet/TBET/TBLYM |
| F12 | Hs.29344 | TICAM1 | Toll-like receptor adaptor molecule 1 | IIAE6/MyD88-3/PRVTIRB/TICAM-1/TRIF |
| G01 | Hs.654532 | TLR1 | Toll-like receptor 1 | CD281/TIL/TIL. LPRS5/rsc786 |
| G02 | Hs.519033 | TLR2 | Toll-like receptor 2 | CD282/TIL4 |
| G03 | Hs.657724 | TLR3 | Toll-like receptor 3 | CD283/IIAE2 |
| G04 | Hs.174312 | TLR4 | Toll-like receptor 4 | ARMD10/CD284/TLR-4/TOLL |
| G05 | Hs.604542 | TLR5 | Toll-like receptor 5 | MELIOS/SLE1/SLEB1/TIL3 |
| G06 | Hs.575090 | TLR6 | Toll-like receptor 6 | CD286 |
| G07 | Hs.659215 | TLR7 | Toll-like receptor 7 | TLR7-like |
| G08 | Hs.660543 | TLR8 | Toll-like receptor 8 | CD288 |
| G09 | Hs.87968 | TLR9 | Toll-like receptor 9 | CD289 |
| G10 | Hs.241570 | TNF | Tumor necrosis factor | DIF/TNF-alpha/TNFA/TNFSF2 |
| G11 | Hs.591983 | TRAF6 | TNF receptor-associated factor 6 | MGC:3310/RNF85 |
| G12 | Hs.75516 | TYK2 | Tyrosine kinase 2 | IMD35/JTK1 |
| **H01** | **Hs.520640** | **ACTB** | **Actin, beta** | **BRWS1/PS1TP5BP1** |
| **H02** | **Hs.534255** | **B2M** | **Beta-2-microglobulin** | **-** |
| **H03** | **Hs.592355** | **GAPDH** | **Glyceraldehyde-3-phosphate dehydrogenase** | **G3PD/GAPD/HEL-S-162eP** |
| **H04** | **Hs.412707** | **HPRT1** | **Hypoxanthine phosphoribosyltransferase 1** | **HGPRT/HPRT** |
| **H05** | **Hs.546285** | **RPLP0** | **Ribosomal protein, large, P0** | **L10E/LP0/P0/PRLP0/RPP0** |
| **H06** | **N/A** | **HGDC** | **Human Genomic DNA Contamination** | **HIGX1A** |
| **H07** | **N/A** | **RTC** | **Reverse Transcription Control** | **RTC** |
| **H08** | **N/A** | **RTC** | **Reverse Transcription Control** | **RTC** |
| **H09** | **N/A** | **RTC** | **Reverse Transcription Control** | **RTC** |
| **H10** | **N/A** | **PPC** | **Positive PCR Control** | **PPC** |
| **H11** | **N/A** | **PPC** | **Positive PCR Control** | **PPC** |
| **H12** | **N/A** | **PPC** | **Positive PCR Control** | **PPC** |
