# Supplementary figures and images for: Streptococcus thermophilus alters the expression of genes associated with innate and adaptive immunity in human peripheral blood mononuclear cells
Source: PLoS One. 2020 Feb 11;15(2):e0228531. doi: 10.1371/journal.pone.0228531 (PMC7012395; doi:10.1371/journal.pone.0228531)

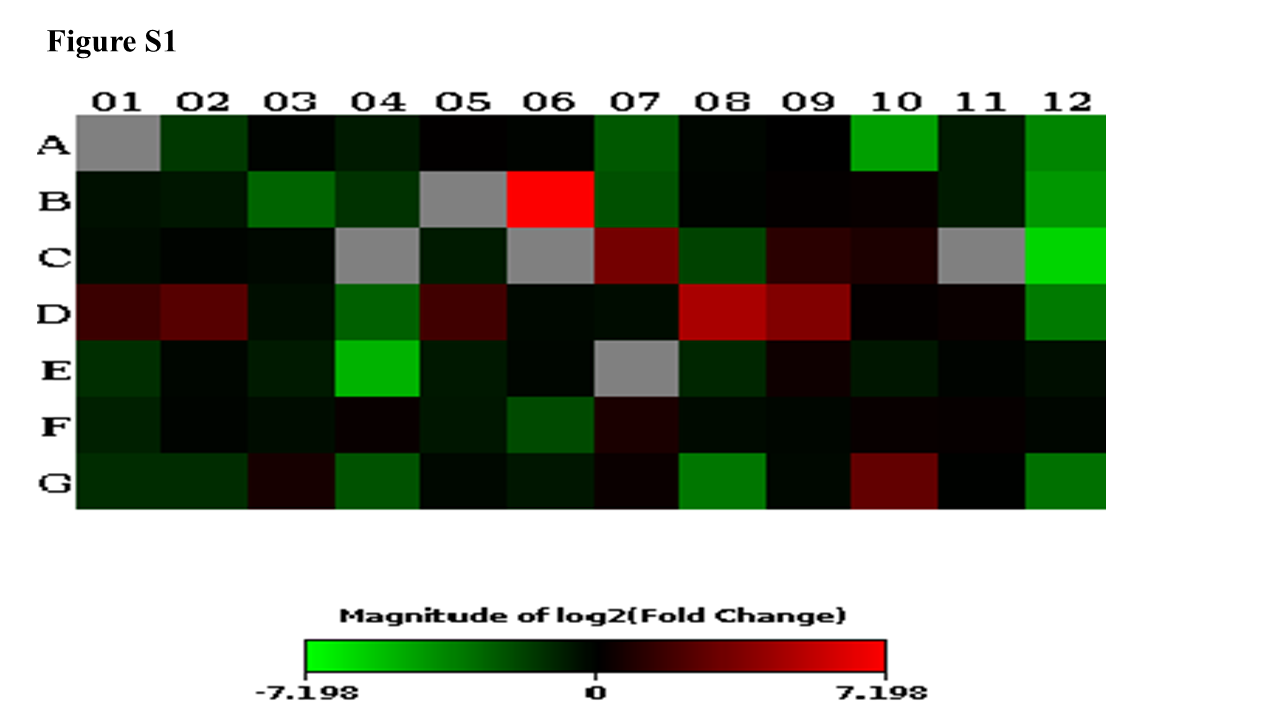

Supplement: S1 Fig — Green represents down regulated genes to red represents upregulated genes. (DOCX) [file pone.0228531.s002.docx]
